# Epidemiology and spatial distribution of bluetongue virus in Xinjiang, China

**DOI:** 10.7717/peerj.6514

**Published:** 2019-02-22

**Authors:** Jun Ma, Xiang Gao, Boyang Liu, Hao Chen, Jianhua Xiao, Hongbin Wang

**Affiliations:** Department of Veterinary Surgery, Northeast Agricultural University, Harbin, China

**Keywords:** Spatial distribution, Bluetongue, Spatial autocorrelation, MaxEnt

## Abstract

Bluetongue (BT) is a non-contagious disease affecting domestic and wild ruminants. Outbreaks of BT can cause serious economic losses. To investigate the distribution characteristics of bluetongue virus (BTV), two large-scale censuses of BTV prevalence in Xinjiang, China were collected. Spatial autocorrelation analysis, including global spatial autocorrelation and local spatial autocorrelation, was performed. Risk areas for BTV occurrence in Xinjiang were detected using the presence-only maximum entropy model. The global spatial autocorrelation of BTV distribution in Xinjiang in 2012 showed a random pattern. In contrast, the spatial distribution of BTV from 2014 to 2015 was significantly clustered. The hotspot areas for BTV infection included Balikun County (*p* < 0.05), Yiwu County (*p* < 0.05) and Hami City (*p* < 0.05) in 2012. These three regions were also hotspot areas during 2014 and 2015. Sheep distribution (25.6% contribution), precipitation seasonality (22.1% contribution) and mean diurnal range (16.2% contribution) were identified as the most important predictors for BTV occurrence in Xinjiang. This study demonstrated the presence of high-risk areas for BTV infection in Xinjiang, which can serve as a tool to aid in the development of preventative countermeasures of BT outbreaks.

## Introduction

Bluetongue (BT) is a non-contagious disease affecting ruminant and camelid species (Hofmann et al., 2008). BT, caused by Bluetongue virus (BTV), is a vectorborne disease transmitted between ruminant hosts by blood-feeding midges of the *Culicoides spp*. Hosts of BTV infection are domestic and wild ruminants, including sheep, goats, cattle and deer. Among these, goats and cattle are often considered as asymptomatic reservoir hosts (Maclachlan, 1994) or sub-clinically affected (Maclachlan et al., 2009). Severe clinical signs are often seen in certain breeds of sheep, European fine wool and mutton breeds for example (Maclachlan et al., 2009). The most commonly observed clinical signs include fever, hyperemia in nasal and oral mucosa, edema in the lip, ulcers of the oral mucosa, cyanosis of the tongue, and skeletal muscle deformation. Cyanotic tongues are the most obvious characteristic that aid in differentiation from other diseases.

Within the *Orbivirus* genus in the *Reoviridae* family, BTV is the prototype member (Coetzee et al., 2012). Currently, 26 serotypes of BTV (BTV 1 to BTV 26) have been serologically identified (Maan et al., 2011). The BTV genome consists of 10 linear dsRNA genome segments (Verwoerd, Louw & Oellermann, 1970), which encode seven structural (VP1-7), and five non-structural proteins (NS1, NS2, NS3, NS3/A and NS4) (Belhouchet et al., 2011; Van Dijk & Huismans, 1988). Differences of the outer capsid proteins, particularly VP2 (Huismans & Erasmus, 1981), determine which of the 26 serotypes the virus belongs.

Late in the 18th century, BT was first reported officially in Cape of Good Hope, South Africa. After a systematic clinical study, Spreull named the disease “BT” for the first time in 1905, with reference to the characteristic cyanotic tongues of the infected sheep (Spreull, 1905). In 1943, an outbreak of BT occurred in Cyprus, which is believed to be the first occurring outside of Africa (Gambles, 1949). Since that time, BT has subsequently occurred in many regions of the world, with Antarctica being the only continent free of BTV infection for now (Maclachlan et al., 2009). In China, BTV has become widely distributed throughout the mainland since it was first reported in Yunnan Province in 1979. At present, 11 serotypes of BTV have been isolated in China (Yang et al., 2016; Zhang et al., 1999), with BTV-1 and BTV-16 being the most commonly isolated serotypes (Lee et al., 2011; Zhang et al., 1999).

Outbreaks of BT can create serious economic consequences. Extensive measures are required to control the spread of the virus among infected and cohoused livestock. It is estimated that economic losses resulting from a BT outbreak in 1996 totaled more than $3 billion USD worldwide (Tabachnick, 1996). Thus, BT was included on the World Organisation of Animal Health (OIE) list of notifiable diseases in the mid 1960s, and is classified as a Class A disease of concern in China as well. At present, there are no effective treatments for the disease. Thus, measures to prevent and control BT outbreaks are of critical importance.

In this study, data from two recent large-scale sampling investigations (2012 and 2014–2015) in Xinjiang Province, China were further analyzed. To investigate the distribution characteristics of BTV, a global and local spatial autocorrelation analysis was performed. Risk areas for BTV occurrence in Xinjiang were detected using the presence-only maximum entropy (MaxEnt) model. This work presents the risk zones of BT in Xinjiang Province, which may subsequently provide useful information for the development of effective strategies for the prevention and control of BT outbreaks.

## Materials and Methods

### Study area

**Figure 1:**
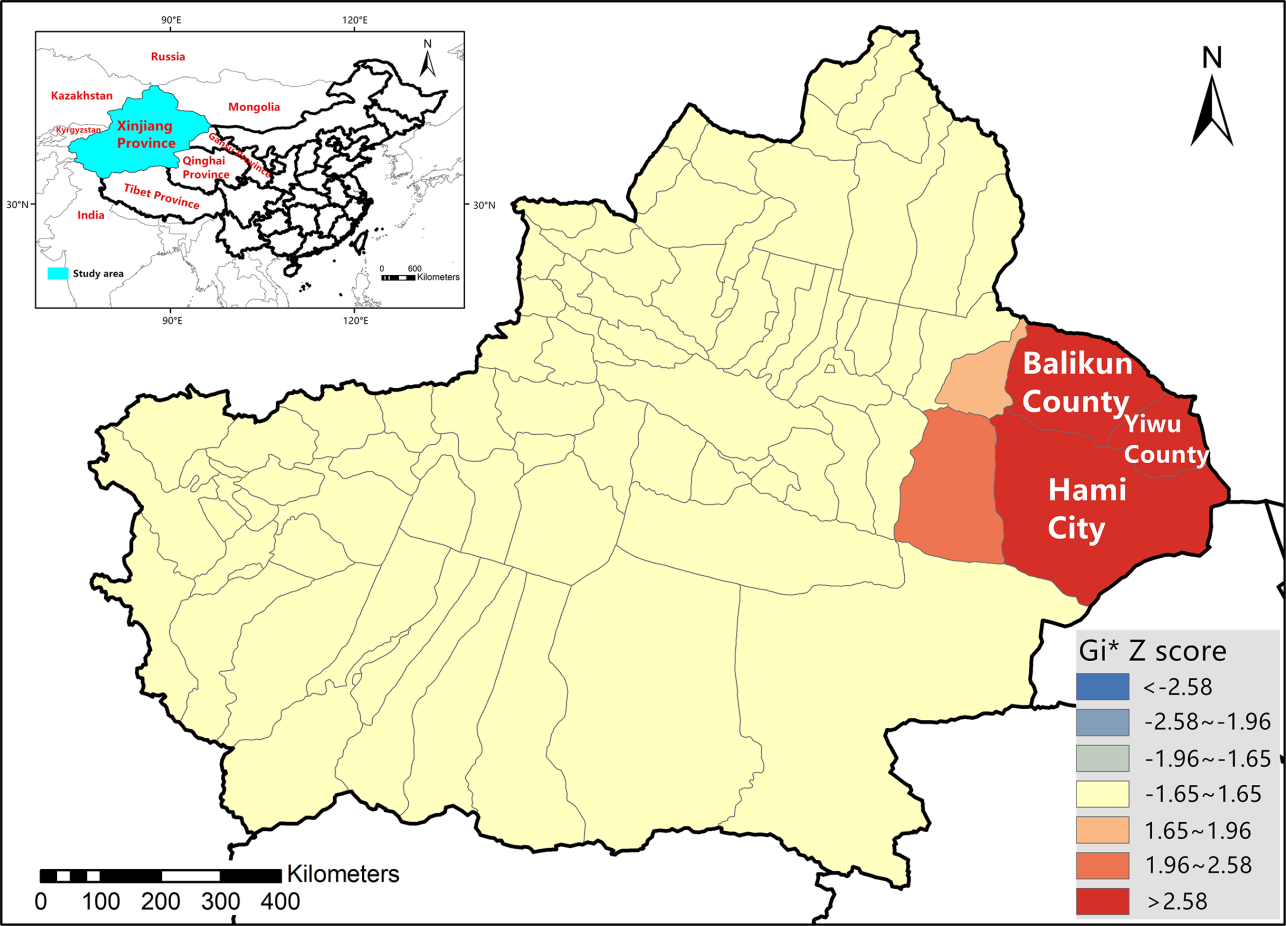
Location of Xinjiang Province on the map of China and bluetongue virus hotspot analysis in Xinjiang Province in 2012.

The Xinjiang Uygur Autonomous Region is located along the northwestern border of China (Fig. 1), extending from latitude 34°22′ to 49°10′N and longitude 73°40′ to 96°23′E. The region has a temperate continental climate. According to the data released by the National Bureau of Statistics of the People’s Republic of China (http://www.stats.gov.cn/), Xinjiang is the second largest province in terms of sheep and goat production in China.

### Collection and processing of samples

In 2012, a total of 1,441 blood samples were collected from sheep and goats among seven prefectures (Bayingolin Mongolian Autonomous Prefecture, Hotan Prefecture, Aksu Prefecture, Kashi Prefecture, Changji Hui Autonomous Prefecture, Ili Kazak Autonomous Prefecture, Hami City) including seven counties (cities) (Yuli County, Hotan County, Wensu County, Taxkorgan County, Mori County, Qapqal County, Hami City) in Xinjiang, China. Between 2014 and 2015, a total of 2,135 sheep and goat blood samples were collected among eight prefectures (Kumul Prefecture, Changji Hui Autonomous Prefecture, Urumchi, Turpan Prefecture, Ili Kazakh Autonomous Prefecture, Bayingolin Mongol Autonomous Prefecture, Hotan Prefecture, Kashgar Prefecture) including 15 counties (cities) (Balikun County, Yiwu County, Hami City, Qitai County, Jimsar County, Urumqi City, Shanshan County, Turpan City, Toksun County, Yining County, Hejing County, Heshuo County, Yuli County, Hotan City, Taxkorgan County) in Xinjiang, China. All samples were tested at the Institute of Veterinary Medicine, Xinjiang Academy of Animal Science. Competitive enzyme linked immunosorbent assay (c-ELISA) was used to test for serological evidence of BTV infection. The serum test kit for BTV was kindly provided by the Yunnan Key Laboratory of Tropical and Subtropical Animal Virus Diseases.

The instability caused by variance of BTV apparent prevalence might over inflate the estimate of BTV prevalence. To account for this, Empirical Bayes smoothing was performed in openGeoDa (Anselin & Mccann, 2009) before mapping (Barro et al., 2015; Leyland & Davies, 2005).

### Environmental data collection

To characterize the environmental requirements for BTV to be present, 21 environmental factors that may influence BT occurrence were initially selected (García-Bocanegra et al., 2018; Remya, Ramachandran & Jayakumar, 2015). Environmental factors included 19 bioclimatic variables (Bio 1-Bio 19) and gridded sheep and goat densities (SD and GD). The 19 bioclimatic variables obtained from WorldClim (http://www.worldclim.org/), represent annual trends, seasonality and limiting or extreme environmental factors. Two animal distribution variables were obtained from Livestock Geo-Wiki (https://livestock.geo-wiki.org/home-2/), and represent sheep and goat densities. All the environmental parameters were converted to ASCII raster grids and preprocessed to a spatial resolution of 30 arc-seconds (ca. one km^2^ at ground level).

### Spatial autocorrelation analysis

The distribution of a phenomenon is presented as either a clustered, dispersed, or random pattern within a given space. Spatial autocorrelation analysis was used to investigate geographic patterns of BTV distribution in the Xinjiang Province. BTV positive rate was taken as the attribute value. Both local and global spatial autocorrelations were used to analyze the datasets.

#### Global spatial autocorrelation analysis

A global spatial autocorrelation was applied to analyze the distribution of BTV where all counties were seen as a whole. Global Moran’s I measured the spatial autocorrelation of county locations and the BTV positive rate. Global Moran’s I ranges from −1 to 1, which correspond to highly dispersed and highly clustered distributions, respectively. These parameters were calculated as follows (Cliff & Ord, 1973):

$${\rm{Moran's}} \ I = {{n\mathop \sum \nolimits_i \mathop \sum \nolimits_j {W_{ij}}\left( {{X_i} - \bar X} \right)\left( {{X_j} - \bar X} \right)} \over {\mathop \sum \nolimits_i \mathop \sum \nolimits_j {W_{ij}}{{\left( {{X_j} - \bar X} \right)}^2}}}$$

where:

*X_i_* = the BTV positivity rate in the *i*th county;
$\overline {X}$ = the mean of the BTV positivity rate in all counties within Xinjiang Province;
*X_j_* = the BTV positivity rate in the *j*th county;
*W_ij_* = a weight parameter for the pair of counties *i* and *j* that represents proximity;
*n* = the number of counties in Xinjiang Province.

#### Local spatial autocorrelation analysis

Local spatial autocorrelation was applied to explore the BTV distribution mode within a particular county. The local Getis-Ord *G*_i_^*^ statistic (Hinman, Blackburn & Curtis, 2006) and its *Z*-value were calculated to test for statistical significance of BTV local autocorrelation values. If *G*_i_^*^ > 0 and *Z* > 1.96, the county would be considered as a hotspot area, indicating that BTV distribution within this province were spatially clustered with a significance level of 99% (*p* < 0.01). Getis-Ord *G*_i_^*^ was calculated as follows (Getis & Ord, 2010):

$$G_i^* = {{\mathop \sum \nolimits_{j = 1}^n {W_{ij}}{X_j} - \bar X\mathop \sum \nolimits_{j = 1}^n {W_{ij}}} \over {S\sqrt {{{n\mathop \sum \nolimits_{j = 1}^n W_{ij}^2 - {{\left( {\mathop \sum \nolimits_{j = 1}^n {W_{ij}}} \right)}^2}} \over {n - 1}}} }}$$

where:

*X_i_* = the BTV positivity rate in the *j*th county;
*W_ij_* = a weight parameter for the pair of counties *i* and *j* that represents proximity;
$\overline {X}$ = the mean of the BTV positivity rate in all counties within Xinjiang;
*n* = the number of counties in Xinjiang;
*S* = the standard deviation.

### Maximum entropy modeling

Risk areas for BTV infection in Xinjiang were detected using the presence-only MaxEnt ecological niche model (Phillips, Anderson & Schapire, 2006). BTV positive livestock samples were used as proxies for the vector-borne BTV to model the virus presence. Correlation among environmental factors was assessed using Spearman’s rank correlation coefficient to avoid variable multicollinearity that can result in model over-fitting (Graham, 2003). A cross-correlation value less than 0.75 was used as the cut-off threshold to exclude highly correlated variables (Zhang et al., 2012). Variables considered co-linear were excluded and nine variables were selected as evaluator variables.

The MaxEnt model used BTV positive locations in 2012, 2014 and 2015 as presence data and 10,000 randomly chosen background points as “Pseudo-Absence” data. To reduce environmental bias resulting from sampling bias introduced from spatially clustered occurrences, all the locality data of 164 BTV positive samples were rarefied at one km^2^. A total of 112 occurrence locality data remained for model development after rarefying the database using the *Spatially Rarefy Occurrence Data for SDMs* tool (Brown, Bennett & French, 2017). A total of 75% of the BTV occurrence locations were used as the training set for model calibration, and 25% were used as the testing set for model evaluation. A regularization value of one was used to avoid over fitting of the test data. Area under the curve (AUC) of the receiver operating characteristic plot was calculated to evaluate the produced model (Phillips, Anderson & Schapire, 2006). AUC ranges from zero to one, with one indicating perfect discrimination (Fielding & Bell, 1997). The importance of each variable was assessed using the Jackknife test and percent contribution. The potential species distribution map had a series of values from zero to one which indicated low potential to high potential. These values were regrouped into four classes of potential habitats with high potential (>0.421), moderate potential (0.296–0.421) and low potential (<0.296) based on 10th percentile presence threshold (0.296) (Hannah & Edwin, 2012) and the maximum training sensitivity plus specificity (0.421) (Bean, Stafford & Brashares, 2012).

Maximum entropy models were developed using MaxEnt version 3.2.0. Final outputs of the model predictions were visualized in ArcGIS 10.2.

### Ethics statement

Ethics Committee approval was obtained from the Laboratory Animal Ethics Committee of Northeast Agricultural University to the commencement of the study.

## Results

### C-ELISA for BTV

During 2012, a total of 1,441 sheep and goat blood samples were collected to assess the prevalence of BT. A total of 19 samples were determined to be positive for BTV (1.32%). The rate of BTV positive samples was the highest in Yuli County, at 3.33%. Qapqal County was found to be free of BTV in 2012. The detailed data are presented in Table 1.

**Table 1:** Result of c-ELISA for BTV in Xinjiang in 2012.

Prefectures	Counties (cities)	Number of samples	Number of positive samples	Positive rate%
Bayingolin Mongolian Autonomous Prefecture	Yuli County	120	4	3.33
Hotan Prefecture	Hotan County	137	3	2.19
Aksu Prefecture	Wensu County	218	2	0.92
Kashi Prefecture	Taxkorgan County	273	6	2.20
Changji Hui Autonomous Prefecture	Mori County	200	2	1.00
Ili Kazak Autonomous Prefecture	Qapqal County	100	0	0.00
Hami City	Hami City	393	2	0.51
In total		1,441	19	1.32

In 2014 and 2015, A total of 2,135 sheep and goat blood samples were collected to determine the prevalence of BT. There were 145 samples found to be BTV positive (6.79%). The rate of BTV positive animals in Hejing County was the highest at 15.33%. Turpan Prefecture was observed to be free of BTV. The detail information is shown in Table 2.

**Table 2:** Result of c-ELISA for BTV in Xinjiang from 2014 to 2015.

Prefectures	Counties (cities)	Number of samples	Number of positive samples	Positive rate%
Kumul Prefecture	Balikun County	115	5	4.35
	Yiwu County	98	4	4.08
	Hami City	156	8	5.13
Changji Hui Autonomous Prefecture	Qitai County	125	14	11.20
	Jimsar County	133	8	6.02
Urumchi	Urumqi City	140	12	8.57
Turpan Prefecture	Shanshan County	120	0	0.00
	Turpan City	136	0	0.00
	Toksun County	122	0	0.00
Ili Kazakh Autonomous Prefecture	Yining County	132	7	5.30
Bayingolin Mongol Autonomous Prefecture	Hejing County	137	21	15.33
	Heshuo County	215	19	8.84
	Yuli County	235	33	14.04
Hotan Prefecture	Hetian City	127	11	8.66
Kashgar Prefecture	Taxkorgan County	144	3	2.08
In total		2,135	145	6.79

### Spatial autocorrelation analysis

#### Global spatial autocorrelation analysis

The results of the global spatial autocorrelation analysis are presented in Table 3. The 2012 distribution of BTV in Xinjiang followed a random pattern, but was significantly clustered in 2014–2015.

**Table 3:** Global spatial autocorrelation analysis of BTV in the Xinjiang Province.

Year	Global Moran’s I	*Z* score	*p-*value
2012	0.0002	0.3708	0.7108
2014–2015	0.0526	1.8150	0.0695

#### Local spatial autocorrelation analysis

**Figure 2:**
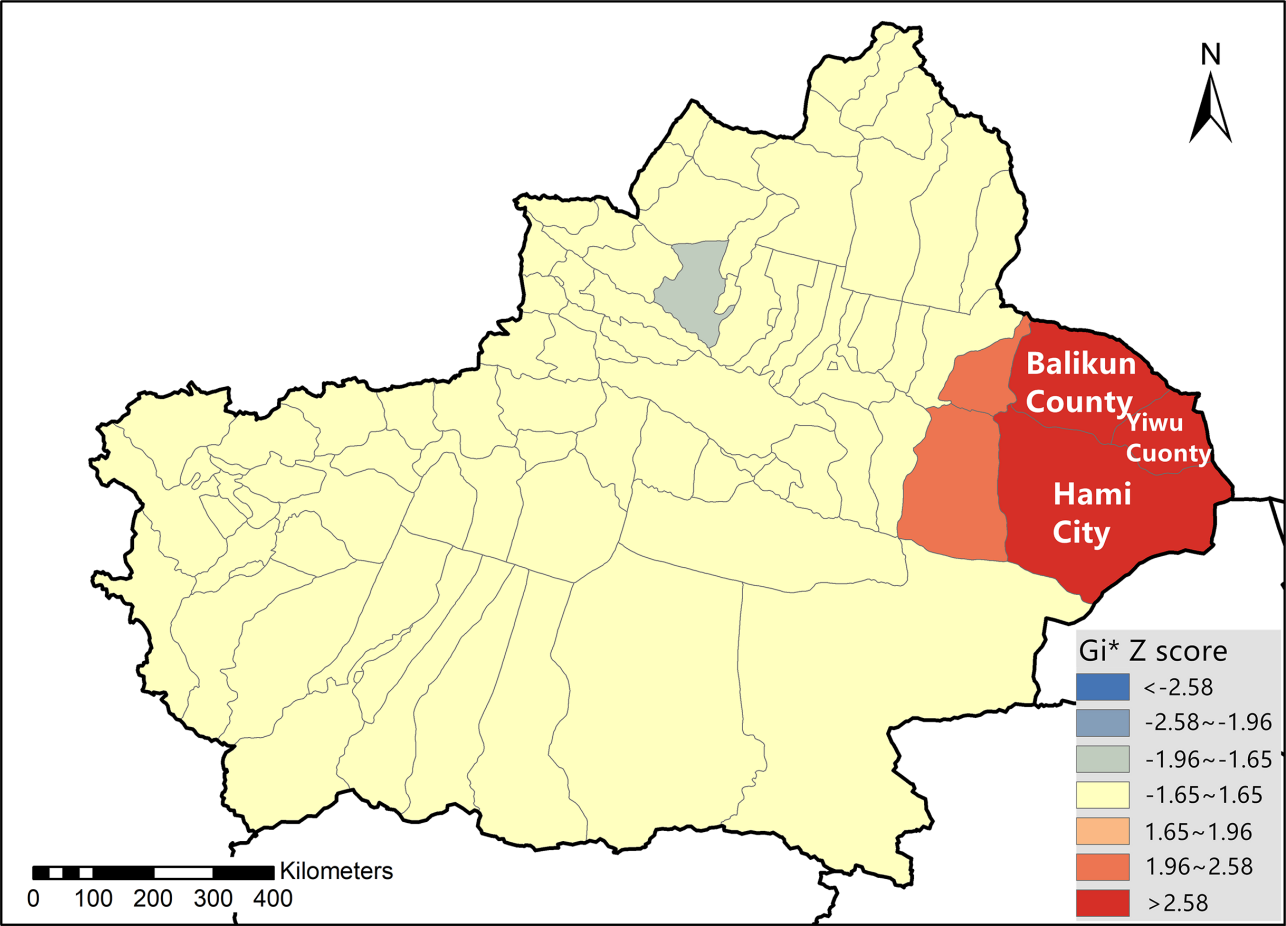
Bluetongue virus hotspot analysis in Xinjiang Province during 2014 and 2015.

The results of the 2012 BTV hotspot analysis in Xinjiang Province are presented in Fig. 1 and Table S1. As is demonstrated in the figure, Balikun County (*Z* = 3.1256, *p* = 0.0018), Yiwu County (*Z* = 4.7139, *p* < 0.001) and Hami City (*Z* = 4.1515, *p* < 0.001) were hotspot areas for BTV infection in 2012. Figure 2 showed the result of the hotspot analysis of BTV distribution in Xinjiang from 2014 to 2015. As shown in the figure, during 2014 and 2015, Balikun County (*Z* = 4.0818, *p* = 0.0018), Yiwu County (*Z* = 5.6000, *p* < 0.001) and Hami City (*Z* = 4.9151, *p* < 0.001) were considered to be BTV infection hotspot areas. Table S1 presented *G*_i_* *Z* scores and *p-*values of the hotspot areas.

#### MaxEnt modeling

**Figure 3:**
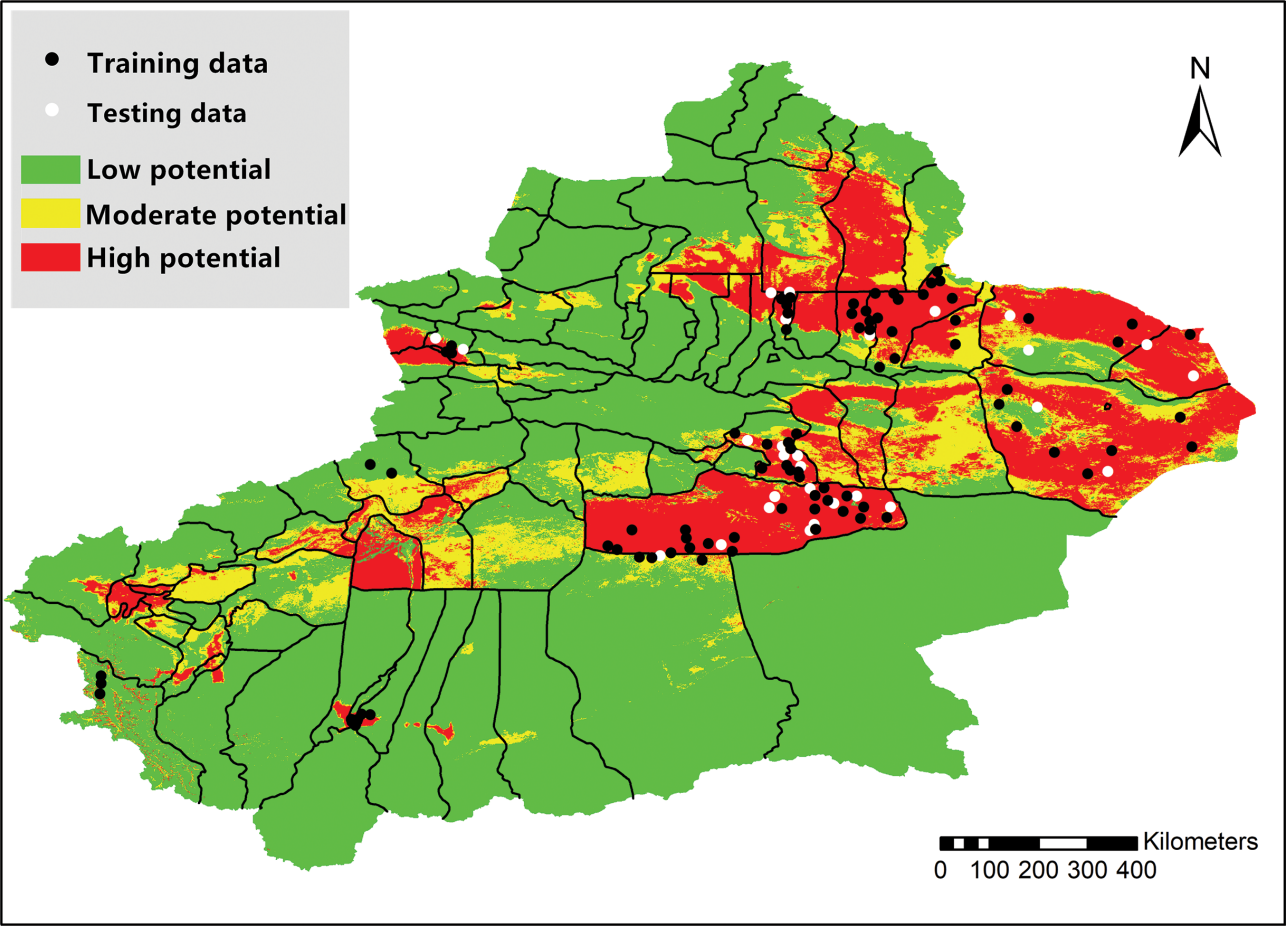
Map of Xinjiang Province showing high-risk areas for bluetongue virus occurrence.

Area under the curve score for the training data was 0.880, indicating that the approach fit the training data fairly well. AUC score for the test data was 0.876 (SD = 0.029), also indicating that the model performed well. As is shown in Table 4, five variables, including sheep distribution (25.6% contribution), precipitation seasonality (22.1% contribution), mean diurnal range (16.2% contribution) and isothermality (16.9% contribution), provided over 80% of model contribution. Sheep distribution, precipitation seasonality and mean diurnal range were identified as the most important predictors for BTV occurrence in Xinjiang. Results of Jackknife test and response curves were shown in Figs. S1 and S2. Figure 3 was a representation of the MaxEnt model for BTV. Warmer colors showed areas with better predicted conditions for BTV occurrence, and these areas were identified as “high-risk” areas.

**Table 4:** Environmental variables used in maximum entropy model for bluetongue virus occurrence.

Abbreviation	Description of variables	Unit	Percent contribution	Permutation importance
Bio 2	Mean diurnal range	°C	16.2	22
Bio 3	Isothermality	–	16.9	18.6
Bio 4	Temperature seasonality	(coeff. of variation °C)	8.4	10.2
Bio 6	Minimum temperature of coldest month	°C	5.6	3
Bio 12	Annual precipitation	mm	1.8	4.6
Bio 15	Precipitation seasonality	(coeff. of variation; %)	22.1	11.9
Bio 19	Precipitation of coldest quarter	mm	3.1	7.8
SD	Sheep density	Heads/km^2^	25.6	21.3
GD	Goats density	Heads/km^2^	0.2	0.7

## Discussion

The first case of BT in China was identified in Yunnan Province in 1979, and BTV was isolated after the outbreak. Soon thereafter, cases of BT in Hubei, Anhui, Sichuan, Gansu and Shanxi Provinces were reported. Concurrently, BTV seropositive animals were found in 29 provinces throughout China, including Guangdong, Guangxi, Jiangsu, Xinjiang, and others, indicating a rapid spread of BT throughout the country. A previously published study covering 27 provinces throughout China between 1987 and 1989 indicated a nationwide BTV seroprevalence rate in sheep and goats of 4.73%. In a similar study, the highest seroprevalence was observed in Guangxi Province in 2001, with 31.7% of sheep and goats testing positive for BTV infection. In Inner Mongolia, the BTV seropositivity rates were 11.75% in 2014 and 11.27% in 2015. It has been demonstrated that BT has become widely established in China for several decades.

In Xinjiang Province, China, three large-scale epidemiological surveys were conducted since the initial cases of BTV were confirmed. The first epidemiological survey analyzed a total of 160,671 blood samples collected from sheep, goats, cattle, yaks and deer from 1988 to 1989 to determine rates of seroconversion to BTV. The result confirmed for the first time that BT was widespread in Xinjiang Province, with goats exhibiting the highest rate of seroconversion to BTV. The second epidemiological survey was conducted in 2012. In total, 1,441 blood samples from sheep and goats, as well as 701 from cattle were collected. The observed rate of BTV positive samples was 1.32% (19 in 1,441) in sheep and goats and 0% (zero in 701) in cattle. However, it has been demonstrated that the rate of BTV positive sera collected in Southern Xinjiang is higher than was observed in Northern Xinjiang. The third and most recent epidemiological survey covered the period from 2014 to 2015. During these two years, 2,135 blood samples collected from sheep and goats were tested, and the rate of BTV positive sera was 6.79% (145 in 2,135). The investigation indicated that the epidemic status of BT in Xinjiang remains a significant concern.

Spatial epidemiology plays an important role in the study of infectious diseases in the field of public health. One such method, spatial autocorrelation, has been used widely in epidemic studies (Al-Ahmadi & Al-Zahrani, 2013; Ma et al., 2017; Ratovonirina et al., 2017). According to our research, in 2012, the distribution of BTV was observed to follow a random pattern, considering Xinjiang Province as a whole. However, local spatial autocorrelation analysis showed that Balikun County (*p* = 0.0018), Yiwu County (*p* < 0.001) and Hami City (*p* < 0.001) were hotspots of BTV infection. It has been proposed by others that case clusters which occur randomly also have an effect on the spread of an infectious disease (Jeefoo, Tripathi & Souris, 2011). During 2014 and 2015, both global and local spatial autocorrelation analyses demonstrated a significantly clustered distribution of BTV in Xinjiang, and that Balikun County (*p* < 0.001), Yiwu County (*p* < 0.001) and Hami City (*p* < 0.001) were also hotspot areas.

In 2011, 48 *Culicoides* species (Diptera: Ceratopogonidae) were recorded in Xinjiang, China (Tian, Liu & Jia, 2011). Adult female haematophagous midges of the *Culicoides spp*. are the only known vectors and the only known mode of transmission through which BTV can spread between susceptible ruminant hosts (Toit, 1944). As such, the epidemic distribution of BT is closely related to the activity of the midge vector (Tabachnick, 2004), which is generally distributed between 40°N and 35°S latitude (Gibbs & Greiner, 1994). However, the hotspot areas for BTV infection observed during the study period lie outside of this geographical range, with the northernmost hotspot being located at 45°N latitude. Additionally, the MaxEnt model identified BTV high-risk areas north to 47°N (Fig. 3) in Xinjiang Province. It is possible that this observation is the result of the expansion of the habitat range of the midge vector due to climate change. It has been reported that the distribution of *Culicoides* are predicted to move northwards up to 53°N latitude with changing climatic and environmental conditions (Zuliani et al., 2015).

Maximum entropy calculates the relationship between the presence data and some environmental predictors which were known to be related to the disease (García-Bocanegra et al., 2018). This study successfully built a presence-only MaxEnt model relying on climatic and environmental data. Sheep distribution, precipitation seasonality and mean diurnal range were identified as the most important predictors for BTV occurrence. Areas with sheep density higher than 500 heads/km^2^ were found to be areas of high risk for BTV (Fig. S2). It has been reported that sheep were most susceptible to BTV infection among all the small ruminants (Coetzee et al., 2012). Moreover, a decrease in the variation coefficient of precipitation seasonality resulted in lower risk of BTV presence. Seasonality of BTV occurrence has been investigated, and the infection regularity in different seasons was observed (Ward, 1996). In this study, the relationship between precipitation seasonality and BTV occurrence may be influenced by the extremely arid climate throughout a whole year in Xinjiang Province. The response curve of Bio 2 showed that areas with mean diurnal range of 14 °C were found to be areas of high risk for BTV. The effect of temperature and precipitation on BTV infection has been investigated (Brand & Keeling, 2017), as well as the effects of seasonal and meteorological parameters on the *Culicoides* existence (Ander, Meiswinkel & Chirico, 2012; Racloz et al., 2008; Sanders et al., 2011). However, influence of annual mean temperature and precipitation on BTV occurrence was not observed in our research. As we know, both biological factors (vegetation, human and animal activities etc.) and natural environmental factors (light, temperature, atmospheric gases etc.) have major impacts on the spread of *Culicoides spp*, which is the major transmission mode (Blanda et al., 2018). Thus, these factors can have a significant influence on the geographical distribution of BTV. For example, an association between BT disease diffusion and some landscape features has been reported (Guis et al., 2007).

As is stated above, data of BTV positive blood samples were used as the input data for the MaxEnt model. Using livestock samples to represent vector-borne BTV may cause the result of niche models and cluster maps to not agree. Future studies should be conducted considering more predictors, including vector, vegetation and other environmental and climatic variables unavailable in this study. Furthermore, factors affecting the distribution of haematophagous midges require much consideration.

Although we believe our research is comparably reliable, limitations still exist. The apparent prevalence may differ from the true prevalence caused by serological tests. A low number of positives with a low number of tests will over inflate the estimates of BTV prevalence, although Empirical Bayes smoothing was performed. The sensitivity and specificity of the test should be performed in the future study. Furthermore, the result of MaxEnt and the hotspot maps do not agree perfectly. Maybe it is because BTV positive cases were used as a proxy for the vector when developing niche models. And the sampling biases, including animal movement and low sample numbers, may also lead to different results.

## Conclusions

The global spatial autocorrelation data on the distribution of BTV in Xinjiang in 2012 exhibited a random pattern, which became markedly clustered in 2014–2015. The hotspot areas for BTV infection included Balikun County, Yiwu County and Hami City in 2012. These three regions were also hotspot areas during 2014 and 2015. A BTV suitability map was generated to show the high-risk areas for BTV occurrence in Xinjiang. This study can serve as a tool for the development of preventative countermeasures for future BT outbreaks.

## Supplemental Information

10.7717/peerj.6514/supp-1 Supplemental Information 1 Relative predictive power of different environmental variables based on the jackknife of regularized training gain in MaxEnt models for BTV.

10.7717/peerj.6514/supp-2 Supplemental Information 2 Response curves for environmental predictors in MaxEnt models for BTV.

10.7717/peerj.6514/supp-3 Supplemental Information 3 Results of Empirical Bayes smoothing of BTV apparent prevalence in Xinjiang Province in 2012.

10.7717/peerj.6514/supp-4 Supplemental Information 4 Results of Empirical Bayes smoothing of BTV apparent prevalence in Xinjiang Province during 2014 and 2015.

10.7717/peerj.6514/supp-5 Supplemental Information 5 Result of c-ELISA for BTV in Xinjiang. Raw data.

10.7717/peerj.6514/supp-6 Supplemental Information 6 Results of bluetongue virus hotspot analysis in Xinjiang Province in 2012, 2014 and 2015.

## Additional Information and Declarations

### Data Availability

The following information was supplied regarding data availability: The raw data are available in a Supplemental File.
