# Supplementary figures and images for: Epidemiology and spatial distribution of bluetongue virus in Xinjiang, China
Source: PeerJ. 2019 Feb 22;7:e6514. doi: 10.7717/peerj.6514 (PMC6388665; doi:10.7717/peerj.6514)

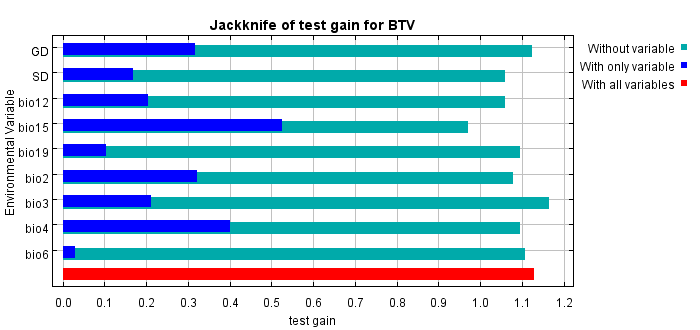

Supplement: Supplemental Information 1 [file peerj-07-6514-s001.png]

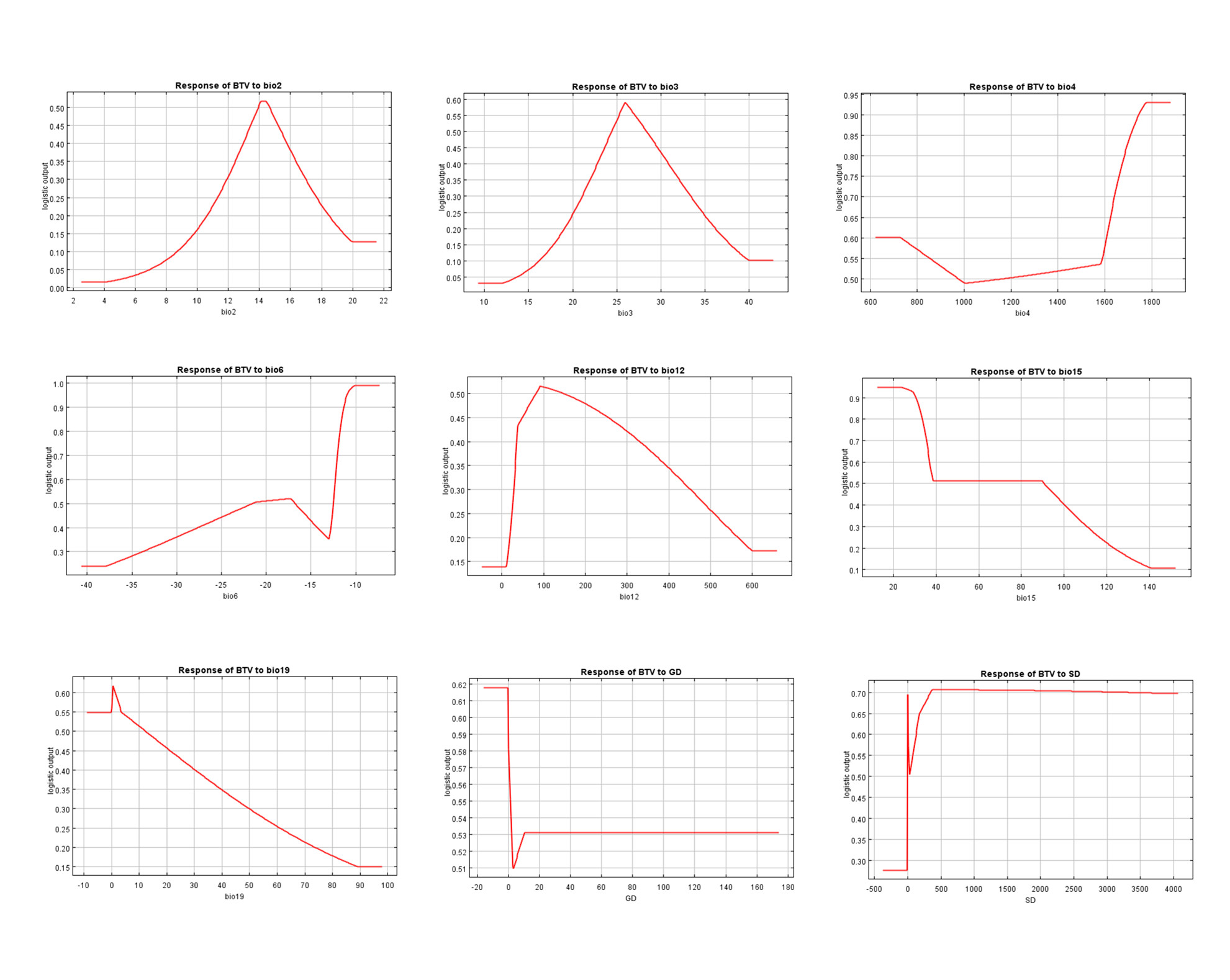

Supplement: Supplemental Information 2 [file peerj-07-6514-s002.png]

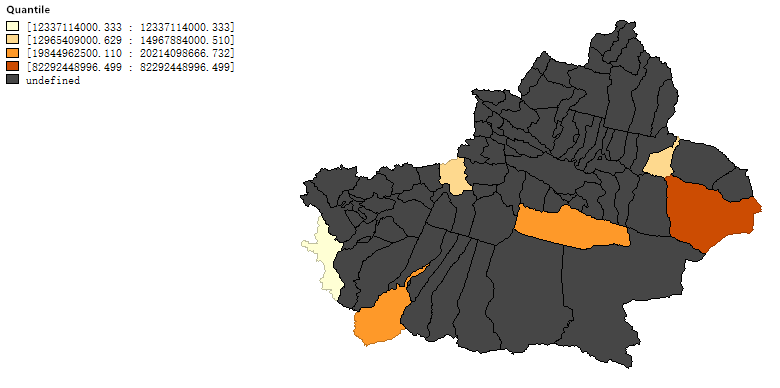

Supplement: Supplemental Information 3 [file peerj-07-6514-s003.png]

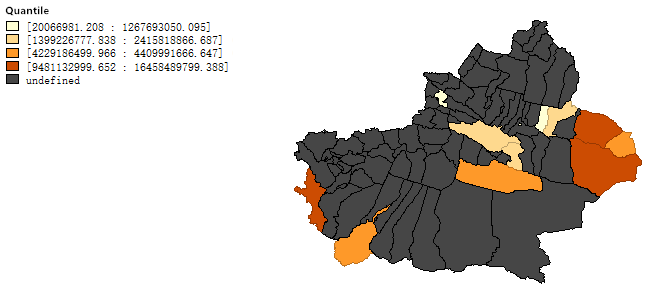

Supplement: Supplemental Information 4 [file peerj-07-6514-s004.png]
