# Supplementary material for: Epidemiology and spatial distribution of bluetongue virus in Xinjiang, China
Source: PeerJ. 2019 Feb 22;7:e6514. doi: 10.7717/peerj.6514 (PMC6388665; doi:10.7717/peerj.6514)
Supplement: Supplemental Information 6 [file peerj-07-6514-s006.docx]

Table S1 Results of bluetongue virus hotspot analysis in Xinjiang Province in 2012, 2014 and 2015.

| Region | 2012 | | 2014-2015 | |
| --- | --- | --- | --- | --- |
|  | Gi^*^Z Score | Gi^*^P Value | Gi^*^Z Score | Gi^*^P Value |
| Balikun County | 3.1256 | 0.0018 | 4.0818 | <0.0001 |
| Yiwu County | 4.7139 | <0.0001 | 5.6000 | <0.0001 |
| Hami City | 4.1515 | <0.0001 | 4.9151 | <0.0001 |
